# 

**Published:** 1856-05

**Authors:** 


PUBLISHED MONTHLY, AT $3 PER ANNUM IN ADVANCE.

### ATLANTA MEDICAL AND SURGICAL JOURNAL.

EDITED BY

**JOSEPH P. LOGAN, M. D.,**

PROFESSOR OF PHYSIOLOGY AND GENERAL PATHOLOGY,

AND

**W. F. WESTMORELAND, M. D.,**

PROFESSOR OF THE PRINCIPLES AND PRACTICE OF SURGERY.

**Pax et scientia, sed veritas sine timore.**

VOL. I. MAY, 1856. NO. IX.

ATLANTA, GEORGIA:

PRINTED BY C. R. HANLEITER AND CO.

1856.

☞ Each Number contains sixty-four pages. POSTAGE.—Under 500 miles, 15 cents a year; over 500 miles 30 cents a year—to be pre-paid quarterly. If not pre-paid, double the above rates.

